# AdaptMap: exploring goat diversity and adaptation

**DOI:** 10.1186/s12711-018-0427-5

**Published:** 2018-11-19

**Authors:** Alessandra Stella, Ezequiel Luis Nicolazzi, Curtis P. Van Tassell, Max F. Rothschild, Licia Colli, Benjamin D. Rosen, Tad S. Sonstegard, Paola Crepaldi, Gwenola Tosser-Klopp, Stephane Joost, the AdaptMap Consortium, Marcel Amills, Paolo Ajmone-Marsan, Francesca Bertolini, Paul Boettcher, Robert Boyle Onzima, Dan Bradley, Diana Buja, Margarita Ema Cano Pereira, Antonello Carta, Gennaro Catillo, Licia Colli, Paola Crepaldi, Alessandra Crisà, Marcello Del Corvo, Kevin Daly, Cord Droegemueller, Solange Duruz, Ahmed Elbeltagi, Ali Esmailizadeh, Olivardo Faco, Taina Figueiredo Cardoso, Christine Flury, Josè Fernando Garcia, Bernt Guldbrandtsen, Aynalem Haile, Jon Hallsteinn Hallsson, Michael Heaton, Vivi Hunnicke Nielsen, Heather Huson, Stephane Joost, James Kijas, Johannes A. Lenstra, Gabriele Marras, Marco Milanesi, Chen Minhui, Muhammad Moaeen-ud-Din, Romy Morry O’Donnell, Ogah Moses Danlami, Joram Mwacharo, Ezequiel Luis Nicolazzi, Isabelle Palhière, Fabio Pilla, Mario Poli, Jim Reecy, Barbara Ann Rischkowsky, Estelle Rochat, Benjamin Rosen, Max Rothschild, Rachel Rupp, Brian Sayre, Bertrand Servin, Kleibe Silva, Tad Sonstegard, Gordon Spangler, Alessandra Stella, Roberto Steri, Andrea Talenti, Flavie Tortereau, Gwenola Tosser-Klopp, Elia Vajana, Curtis P. Van Tassell, Wenguang Zhang

**Affiliations:** 1 Istituto di Biologia e Biotecnologia Agraria, Consiglio Nazionale delle Ricerche, Milan, Italy; 2 Fondazione Parco Tecnologico Padano, Lodi, Italy; 3 Animal Genomics and Improvement Laboratory, United States Department of Agriculture, Agricultural Research Service, Beltsville, MD USA; 4 Department of Animal Science, Iowa State University, Ames, IA USA; 5 Università Cattolica del S. Cuore, Piacenza, Italy; 6 Recombinetics Inc., St. Paul, MN USA; 7 Dipartimento di Medicina Veterinaria, University of Milan, Milan, Italy; 8 GenPhySE, INRA, Université de Toulouse, INPT, ENVT, 31326 Castanet Tolosan, France; 9 Laboratory of Geographic Information Systems, École Polytechnique Fédérale de Lausanne, Lausanne, Switzerland

## International initiatives on goat genetics and genomics

Goats are bred worldwide and present in a wide variety of production environments. Local breeds, which are well adapted to a range of agro-ecological conditions, contribute to ensuring the sustainability of livestock farming in marginal and difficult areas in both developed and developing countries. Compared to other livestock species, goats have been domesticated in a single region and subject to a limited amount of hybridization between breeds, thus they represent one of the best species for the study of genetic diversity and adaptation.

The International Goat Genome Consortium (IGGC, http://www.goatgenome.org) was created in 2012 with the general goal of increasing the range of genomic tools and publicly available information for the goat. In 2013, the 50 K goat single nucleotide polymorphism (SNP) panel was developed (http://www.goatgenome.org; [3]) by combining whole-genome sequencing and reduced representation libraries from eight breeds/populations from Europe and Asia through the cooperation of the Institut National de la Recherche Agronomique (Inra) in France, Utrecht University in The Netherlands, the Malaysian Agricultural Research and Development Institute (MARDI) in Malaysia, and DNA Landmarks in Canada.

Several large projects took advantage of this newly-developed SNP panel to genotype many goat populations across the world with a range of objectives and hypotheses: genome-wide association analyses across a spectrum of research and production traits, germplasm characterization and diversity studies, and genetic prediction for selection in commercial populations.

The AdaptMap project started as a voluntary consortium in 2014, with the aim of improving coordination among these otherwise independent projects for genotyping, resequencing and phenotyping of goat breeds. AdaptMap was promoted by the International Goat Genome Consortium (IGGC), the African Goat Improvement Network (AGIN), which is a group resulting from the USAID Feed the Future (FtF), the USDA Livestock Improvement Project, the European Union sponsored, 3SR—Sustainable Solutions for Small ruminants and NEXTGEN projects.

## Database

To collect, store and standardize data from many different sources, input tools and a relational database were developed and made accessible to all consortium partners. Provision of data was regulated by a data transfer agreement and uploading was facilitated through a user-friendly graphical interface that allowed users to drag and drop files to the server. A special focus was placed on security to ensure that the data were not compromised in any way.

The first phase of submission and study, to which the series of AdaptMap papers in GSE refer to, accepted data until November 2015. At that point, the AdaptMap raw dataset included 4653 animals sampled from across the world and represented 148 populations, 35 countries and five continents.

**Fig. 1:**
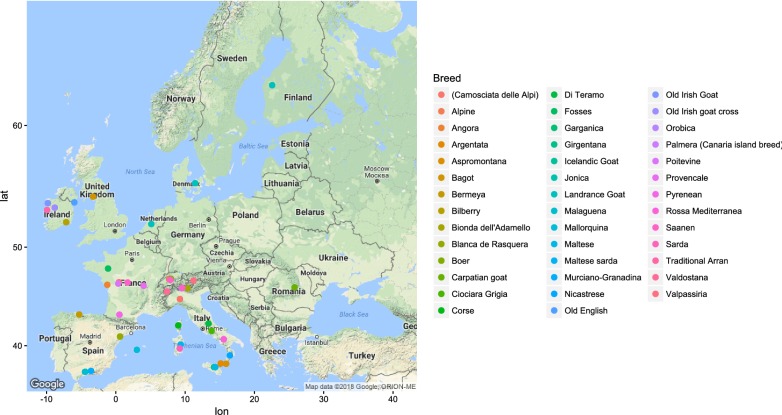
Geographical distribution of the sampled breeds in Europe

**Fig. 2:**
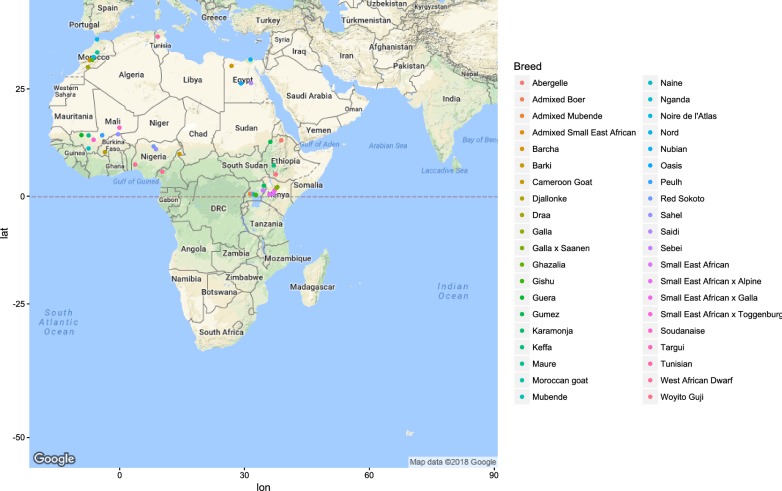
Geographical distribution of the sampled breeds in Africa

**Fig. 3:**
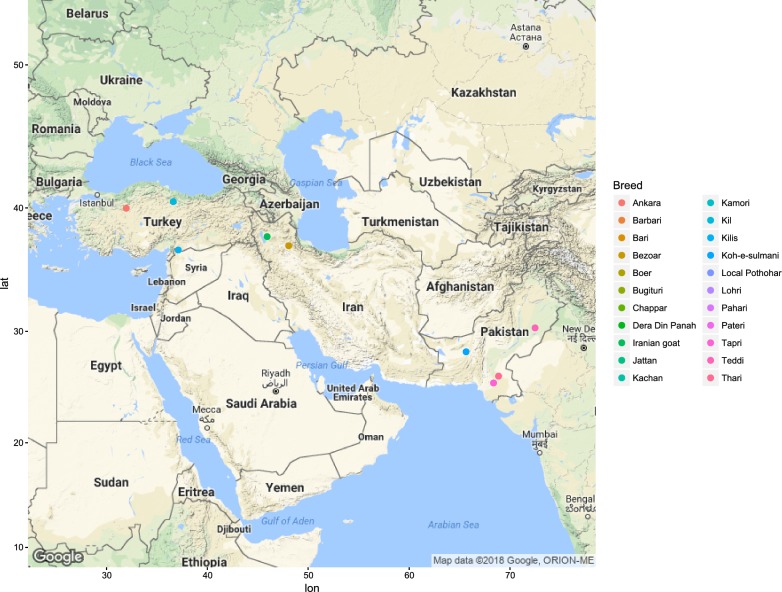
Geographical distribution of the sampled breeds in Asia

**Fig. 4:**
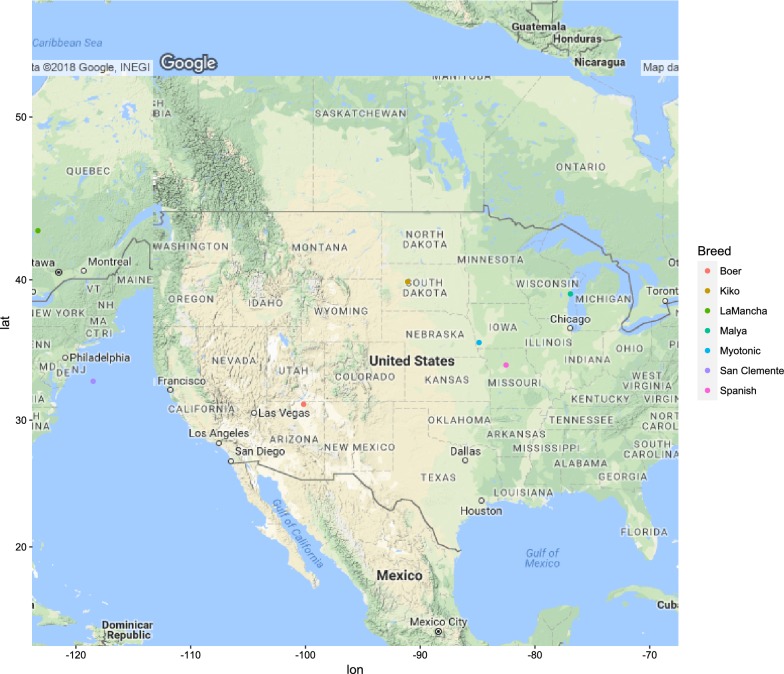
Geographical distribution of the sampled breeds in North America

**Fig. 5:**
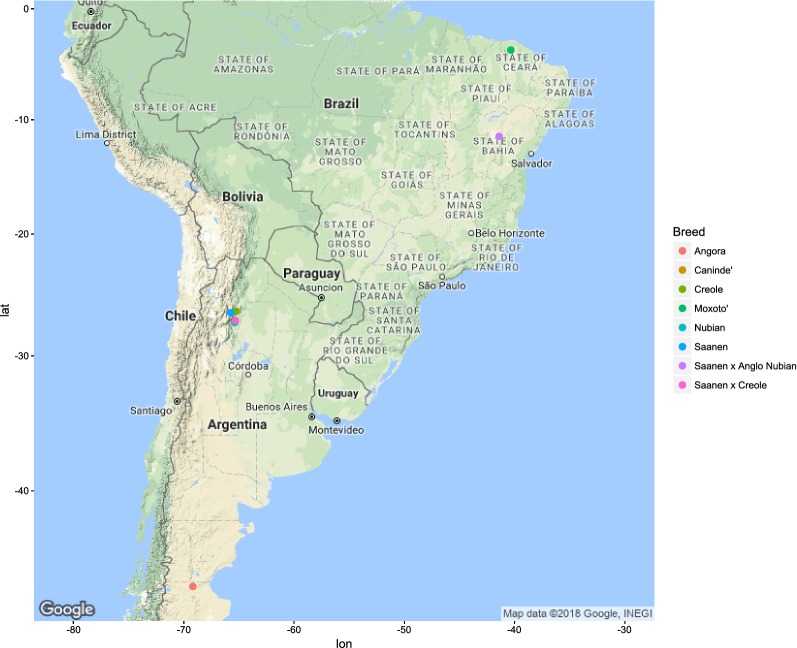
Geographical distribution of the sampled breeds in South America

**Fig. 6:**
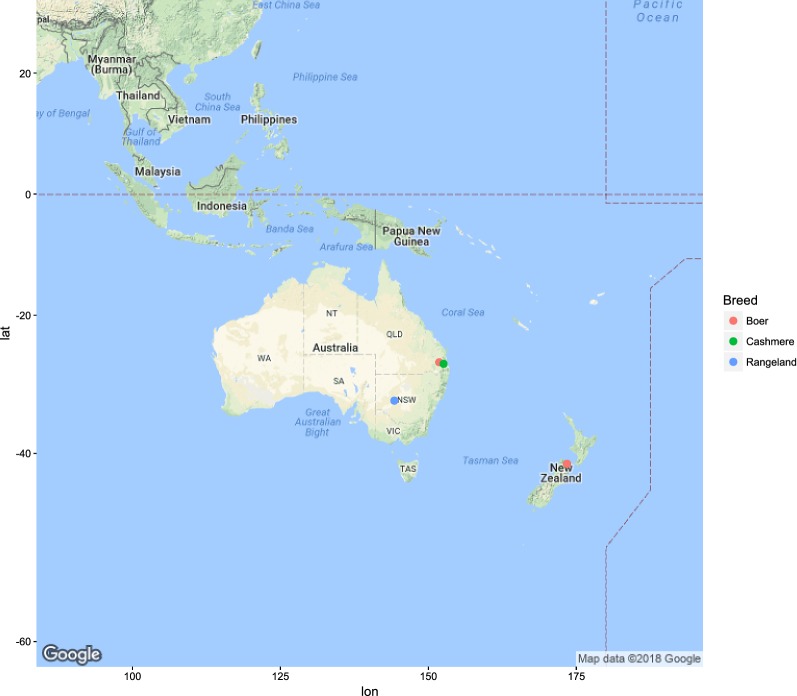
Geographical distribution of the sampled breeds in Oceania

Figures 1, 2, 3, 4, 5 and 6 illustrate the geographical distribution of the sampled breeds. In Europe (Fig. 1), 43 populations were sampled, which comprise commercial international transboundary breeds (e.g. Alpine, Saanen), large populations of locally-adapted transboundary breeds (such as Sarda) and local breeds with small population sizes. A wide range of geo-climatic environments was represented, including the alpine region, productive prairie areas of central Europe, UK and Ireland, the mostly hot and arid regions of southern Spain and small islands and the Carpathians. In Africa (Fig. 2), 70 populations were sampled. The sampling locations comprised a wide range of agro-ecological zones, climates and geographical features. The contribution from the AGIN project was substantial. Both arid climates of North Africa (e.g. Egypt, Tunisia, and Mali) and more humid areas of Sub-Saharan Africa were included. The 21 populations sampled in Western Asia (Fig. 3) included the Bezoar and local goat breeds sampled near the putative centre of domestication (Iranian goat) and along important paths of diversification towards Europe. Pakistani breeds were also represented. The samples from North America (Fig. 4) included both original local breeds, such as La Mancha, exotic breeds such as the New Zealand Kiko, and some populations with peculiar characteristics such as the island goat San Clemente, summed to six breeds. Climates varied from the arid region of Texas to the more temperate northern east coast. South American (Fig. 5) contributions totalled six breeds from Brazil and Argentina, which originate from very different latitudes, including the local Canindè and Moxoto. Finally, Australia and New Zealand (Fig. 6) samples from three breeds were provided, with rangeland uniquely represented in the dataset by sampling sites in the western part of the continent. For 11 breeds, populations sampled in more than one geographical location. In particular, the Boer and Saanen represented seven and six populations, respectively. Samples were available from four Angora populations and three each from Alpine, Landrace and Small East African breeds. Admixed Boer, Malya, Nubian, Toggenburg and West African Dwarf were each sampled from two populations.

The Illumina GoatSNP50 BeadChip [3] that includes 53,347 SNPs was used to genotype all the animals. Prior to analyses described in the papers of this series, marker positions were re-mapped on the new goat reference sequence ARS1 [1].

After populating the database, quality control was performed on the whole set using the Plink 1.9 software [2]. Individuals and SNPs that did not pass the following thresholds were removed: individual genotype call rate higher than 0.96, SNP call rate higher than 0.98, and identity-by-state (IBS) between genotypes (compared pairwise based on all markers) less than 0.99. Moreover, SNPs that were completely monomorphic over the whole dataset were also removed. To identify individuals with high relatedness (e.g. parent–offspring pairs), we calculated the number of Mendelian errors (ME) in pairwise comparisons of all individuals with a locally-developed script. Animals that were most frequently found in pairs with less than 100 ME were removed.

The dataset included 12 crossbred populations: admixed Boer, Galla x Saanen, Matabele cross, admixed Mubende, Old Irish Goat cross, Saanen x ANB, Saanen x Creole, admixed Small East African, Small East African x Alpine, Small East African x Galla, Small East African x Saanen, and Small East African x Toggenburg. These populations were excluded from the analyses for the study of diversity and selection signatures but they do represent an interesting resource for further investigation on adaptation and performances. Similarly, six populations (Bagot, Gishu, Myotonic, Old English, San Clemente, and Tete) were represented by fewer than three animals; for this reason, they were not considered in the study on diversity. Breeds with large populations from multiple locations (Alpine, Angora, Boer, Landrance, Nubian, Saanen) were split to account for the area of origin. Populations with more than 50 individuals were randomly thinned to 50.

## AdaptMap studies

The general scope of the AdaptMap project was to investigate diversity in the goat with a focus on the impact of domestication, adaptation associated with local environmental conditions, and adaptation in response to selection for production systems. The ultimate goal is to enable sustainable breeding by leveraging the use of genomic information. Towards these aims, study groups were organized to make best use of the available genotypic information, and by analytical themes. The first phase of the project addressed by the AdaptMap consortium focused on population genetics analyses and population history, selection signatures, landscape genomics, visible genetic profiles, and the identification of a panel of parentage SNPs. The series of papers published here provide the first reports of the major results from these investigations.

## Major results

Among the major results obtained, perhaps the most striking was the corroboration of prior observations and results, and of our hypothesis that close associations exist between the genomes of goat breeds and geographical measures.

On a large scale, the studied populations could be partitioned quite distinctly into groups according to continental origin. Three major gene pools were clearly identified, corresponding to goats from Europe, Africa and western Asia. Within these three pools, the patterns of variation were consistent with measures of “distance” in geography, human history and common animal husbandry practices. Specifically, the greatest genetic variability was observed for populations from western Asia, near to the putative center of domestication, with relatively little differentiation among breeds within these regions. Within the other two gene pools, patterns of variation obeyed geographical rules, both on “macro” and “micro” levels. On the macro level, both overall genetic variation and similarity with the western Asian populations tended to decrease for populations sampled from regions located further from western Asia.

On the micro level, the variation observed was consistent with expectations associated with recognized paths of human migration out of the Fertile Crescent region. Comparing the European and African gene pools, European breeds tended to be more discreet, which is consistent with the practice of creating breeds through active human-controlled genetic isolation, rather than allowing breeds to form as a result of climatic influence and geographic barriers. Greater gene flow among populations was observed in Africa. Within both these pools, the genetic impacts of particular instances of geographic isolation were clearly observed, inasmuch as populations from Iceland, Madagascar and the Canary and Balearic Islands tended to have longer and more numerous runs of homozygosity. Various putative “hotspots” of runs of homozygosity were observed, and occasionally these hotspots were unique across continental groups. Geography-based signatures of selection were also identified, the strongest of which was associated with mean annual temperature. Some of the signatures of selection observed correspond to signatures that were reported previously in independent analyses of goats.

Finally, a highly informative panel for parentage assessment was developed to assist breeding in goat populations worldwide.

## Next steps

AdaptMap has now completed the first phase of its planned research but the cooperation generated from the shared objectives, collaborations and large accumulated data offer new opportunities to explore the goat genome. The discovery of additional geographical and ecological differences, individual mutations for traits, disease susceptibility and phenotypic differences will help to provide clues into the amazing adaptability of the goat worldwide and its importance to livestock production.
